# Insights into the Genetic Determination of the Autotetraploid Potato Plant Height

**DOI:** 10.3390/genes14020507

**Published:** 2023-02-16

**Authors:** Long Zhao, Meiling Zou, Sirong Jiang, Xiaorui Dong, Ke Deng, Tiancang Na, Jian Wang, Zhiqiang Xia, Fang Wang

**Affiliations:** 1 Academy of Agriculture and Forestry Sciences, Qinghai University, Xining 810016, China; 2 National Key Laboratory of Sanjiangyuan Ecology and Plateau Agriculture and Animal Husbandry, Qinghai University, Xining 810016, China; 3 Hainan Yazhou Bay Seed Laboratory, Sanya Nanfan Research Institute, College of Tropical Crops, Hainan University, Sanya 572025, China

**Keywords:** autotetraploid potato, plant height, whole genome re-sequencing (WGRS), genome-wide association analysis (GWAS), significant SNP

## Abstract

Plant height is an important characteristic, the modification of which can improve the ability of stress adaptation as well as the yield. In this study, genome-wide association analysis was performed for plant height traits in 370 potato cultivars using the tetraploid potato genome as a reference. A total of 92 significant single nucleotide polymorphism (SNP) loci for plant height were obtained, which were particularly significant in haplotypes A3 and A4 on chromosome 1 and A1, A2, and A4 on chromosome 5. Thirty-five candidate genes were identified that were mainly involved in the gibberellin and brassinolide signal transduction pathways, including the *FAR1* gene, *methyltransferase*, *ethylene response factor*, and *ubiquitin protein ligase*. Among them, *PIF3* and *GID1a* were only present on chromosome 1, with *PIF3* in all four haplotypes and *GID1a* in haplotype A3. This could lead to more effective genetic loci for molecular marker-assisted selection breeding as well as more precise localization and cloning of genes for plant height traits in potatoes.

## 1. Introduction

The potato (*Solanum tuberosum* L.), a staple food for 1.3 billion people, was cultivated on 17.5 million hectares and yielded 370.5 million tons in 2019 worldwide [1]. Two thirds of the annual yield is marketed fresh, while the remainder is processed for snack and other industrial food products, including animal feed, adhesives, pharmaceuticals, wood, and textile commodities [2,3]. It contains abundant starch, proteins, sugars, vitamins, minerals, ascorbic acid, carotenoids, and other trace elements required by humans, thereby making it “the most affordable nutritious food.” It originated in the Andes and evolved into a tuber-forming crop with a short sunlight photoperiod, mainly for asexual reproduction [4]. It was introduced to Europe as early as the 15th century and has been cultivated since the 17th century. The major potato-producing countries include China, Russia, India, Ukraine, and the United States. Nowadays, the area of land in developing countries has surpassed that of developed countries. Global potato production is continuously and gradually growing, with multiple countries becoming highly dependent on potatoes for food production.

Plant height is one of the important agronomic traits targeted in crop breeding and cultivation management. Adequately dwarfed plants have greater lodging resistance and can improve the light energy utilization of the crop population, thereby increasing the crop yield [5]. Since the Green Revolution in the 1950s, phenomenal success has been achieved in reducing plant height to consequently increase the actual crop yield [6]. Currently, some genes regulating plant height have been expressed in crop species, including bread wheat [7], rice [8], and other crops. Brassinosteroids (BRs) and gibberellins (GAs) are the known plant height-promoting regulators with the most direct impact, the fewest side effects, and the greatest agronomic potential [9].

GAs, one of the most important plant hormones, is a class of tetracyclic diterpene phytohormones that play important roles in seed germination, root crown elongation, stem elongation, leaf growth, and flowering and fruiting in plants. Currently, the erythromycin synthesis and metabolic pathway of the model plant *Arabidopsis thaliana* have been studied in depth, with its related genes having been cloned and identified [10,11]. The Green Revolution in crops is closely related to GAs metabolic pathway-related genes, which promote stem elongation and plant height. Researchers localized the QTL (MTD1) that regulates plant height and tiller number in a 66-kb interval on chromosome 9 and clarified that LOC_Os09g02650 depends on the GAs biosynthetic pathway to regulate the rice plant height [12]. A previous study found that the DELLA proteins (Dwarf8 and Dwarf9) regulated maize plant height by regulating gibberellin signal transduction [13].

The sixth major phytohormone, BRs, is a class of steroid phytohormones that is vital for plant growth regulation. It is mainly involved in cell wall remodeling and also promotes cell elongation by regulating the expression of genes, thus promoting the growth of plant organs and regulating plant growth and development [14,15]. Consistent with the primary function of BRs to promote cell elongation, mutants defective in BRs biosynthesis or signal transduction display a dwarf phenotype. Conversely, increasing the BRs levels or activity increased the plant size, biomass, and seed yield [9]. Identification of br-deficient dwarf mutants helped identify several BRs biosynthesis-related enzymes in rice, including *OsDwarf2*, *OsDwarf11*, and *br-deficient dwarf mutant-1* (brd1) and brd2 [16,17]. Reduced expression of *ZmDWF1* (the maize homolog of *DIM1/DWF1*) resulted in dwarf maize plants [18]. Additionally, hormones, including auxin [19] and strigolactone [20], can affect plant stalk development via different processes, including sugar metabolism [21], root hair growth [22], and transcription factor regulation [23].

Genome-wide association study (GWAS) is a genome-wide analysis method to decipher the relationship between phenotypic traits and single nucleotide polymorphism (SNP) markers based on the linkage disequilibrium principle [24]. This can identify candidate genes associated with target traits with high resolution and sensitivity [25,26]. GWAS has been widely used in recent years to study the genetic mechanisms of complex traits in various crops. In this study, we subjected 370 cultivars of tetraploid potatoes to whole genome sequencing (WGS) and performed the GWAS analysis for plant height traits to identify significant molecular markers and candidate genes, while also providing molecular markers and genetic resources for potato plant improvement.

## 2. Materials and Methods

### 2.1. Experimental Material Planting and Sources

A total of 370 potato cultivars were collected from the Institute of Biotechnology, Qinghai Academy of Agricultural and Forestry Sciences, China. Among them, 152 were from Peru, 126 from China, 25 from Israel, Russia, Canada, Australia, the USA, the Netherlands, and New Zealand, and 67 from unknown regions (Table S1).

According to the current guidelines for testing plant cultivars for specificity, consistency, and stability in potatoes (GB/T 19557.28-2018), two independent growth cycles (2019 and 2020, planted at the end of April and harvested at the beginning of October) were set up for observation. The test location was set in Huangyuan County, Qinghai Province (36°680′ N, 101°260′ E). It not only has a cool climate and is extremely suitable for potato cultivar breeding and germplasm conservation but also supports trait collection and identification for maintaining and characterizing the potato material and restoring its native habitat conditions. The propagation material for this study was potato tubers, which were 35–50 mm in diameter, unsprouted, healthy in appearance, and free of pests and diseases. The field trials were divided into three plots, with 6–10 potato plants of each variety planted 30 cm apart from each other and 90 cm apart in rows, with a protected row every 15 rows. A randomized complete block design (RCBD) with three repetitions was used to grow the 370 cultivars.

### 2.2. Identification of Plant Height

The plant height data were collected by observing 370 potato plants with reference to the requirements of trait observation in the national standard for potatoes (GB/T 19557.28-2018). Before the potato was mature, we measured the height of the plant, compared it with the height of the standard cultivars (Table 1), and recorded the height index.

### 2.3. Potato DNA Extraction and Sequencing

A month after the potatoes started growing, the young leaves were removed for DNA extraction. The Plant DNA Extraction Kit (QIAGEN) was used to extract DNA from potato cubes. The DNA purity (OD_260/280_ ratio) was measured using a spectrophotometer, whereas the degree of DNA degradation and RNA/protein contamination was analyzed by agarose gel electrophoresis, and DNA concentration was accurately quantified by Qubit.

Whole genome re-sequencing (WGRS) was performed for DNA libraries using DNBSEQ-T7.

### 2.4. Variable Loci Extraction and Statistics

Raw data were tested by the FastQC software (https://github.com/s-andrews/FastQC/ (accessed on 22 March 2022)), while clean reads were obtained using the fastp software by filtering the raw data that passed the test. Then the BWA software [27] was not only used to establish the index file of the tetraploid potato reference genome Q9 (https://ngdc.cncb.ac.cn/biosample/browse/SAMC490813 (accessed on 22 March 2022)) but was also used for sequence comparison of the clean reads of each sample to get the SAM file. Samtools was used [28] to reorder and convert the SAM file into a BAM file, followed by another sorting and the final generation of the index file. Picard was used to mark the duplicates in the Java environment, which were then used to create citations for the new BAM files.

Then the HaplotypeCaller application of the GATK4 [29] software was used to generate each sample GVCF file (GVCF format includes all variant types, including SNPs and Indel, which need to be further filtered). Finally, the GVCF was merged using CombineGVCFs to obtain the total merged GVCF file, followed by GenotypeGVCFs being used to extract genotypes. The filtered VCF files were then obtained using VCFtools [30] with MAF > 0.05 (minimum allele frequency of 0.05, post removal of rare alleles) and HWE > 0.001 (removal of loci that do not satisfy the Hardy-Weinberg equilibrium (*p* < 0.001)). The VCF tools were further used to remove the variant loci with a >50% deletion rate of all materials to obtain the final filtered, high-quality variant loci. The marker density of SNPs was visualized for mapping using CMplot in R.

### 2.5. Genome-Wide Asociation Analysis

GWAS analysis was performed using the high-quality SNPs and indels obtained. Association analysis was performed on the height of the plants using the compressed mixed linear model (MLM) of the R package GAPIT. Manhattan and QQ plots were plotted using the R package (CMplot) to screen the significant SNP loci with a threshold of *p* = 10^−4^, while the type and location of SNPs were identified using the reference genome of Q9.

### 2.6. Candidate Gene Mining and KEGG Enrichment Analysis

Upstream and downstream genes were searched for significant SNP loci, followed by the screening and identification of the final candidate genes. To determine the functions of the candidate genes and their metabolic pathways, the KEGG database was used for functional annotation to (1) systematically analyze the genetic basis of potato plant height development, (2) mine for superior genes for providing candidate genes and analytical ideas for potato plant morphology formation, and (3) serve potato breeding practice. Meanwhile, to further understand the expression pattern of candidate genes, different parts (including flower, leaf, stem, and root) of the highest plant height of potato Q9 were selected for transcriptome sequencing, with the tetraploid potato Q9 being the reference genome to calculate its expression.

The sampling period for RNA-seq sequencing was the flowering stage. After obtaining the raw data of the transcriptome, we used fastqc software to detect the quality and fastp software to filter the qualified raw data. Furthermore, we compared filtered clean data alignment with reference genome using hisat2 software (http://daehwankimlab.github.io/hisat2/ (accessed on 22 March 2022)), then got and converted Sam files to Bam files, and sorted them using Samtools. Finally, we used the Stringtie [31] software to obtain the expression file (FPKM value).

## 3. Results

### 3.1. Analysis of the Diversity of Potato Plant Height

**Figure 1:**
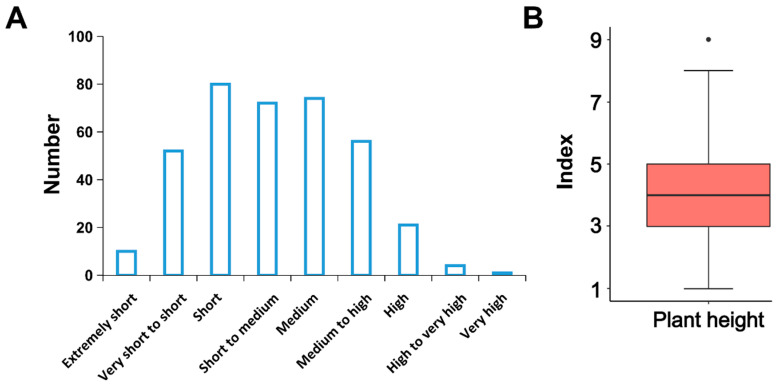
Distribution of the height and boxplot of the potato plant height. (**A**) Plant height distribution of 370 potato cultivars; (**B**) Boxplot of plant height index.

Since the variation was small across environments and years, we took the mean values of the observations from 2019 to 2020 to plot the frequency of their trait distribution (Figure 1A, Table S1). Plant height was dominated by “short”, “dwarf to medium”, and “medium”, with 80 (21.62%), 72 (19.46%), and 74 (20.00%) materials, respectively. The upper, median, and lower limits of plant height were 8, 4, and 1, respectively (Figure 1B), with Q9 being the only plant height index of 9, which was our reference genome cultivar. All plant traits covered all phenotypic states, and all phenotypes conformed to the normal distribution, thus indicating that the population was suitable for GWAS analysis.

### 3.2. Variable Loci Marker Density and Statistical Analysis

The whole genome sequencing of 370 tetraploid potatoes yielded ~9.88 Tb of raw data with an average sequencing depth of ~10 X, ultimately generating 232,581,777 SNPs and Indels. VCFtools helped filter the original VCF file and we obtained 4,986,690 high-quality mutation sites (including 4,535,735 SNPs and 450,955 Indels) (Table 2).

**Figure 2:**
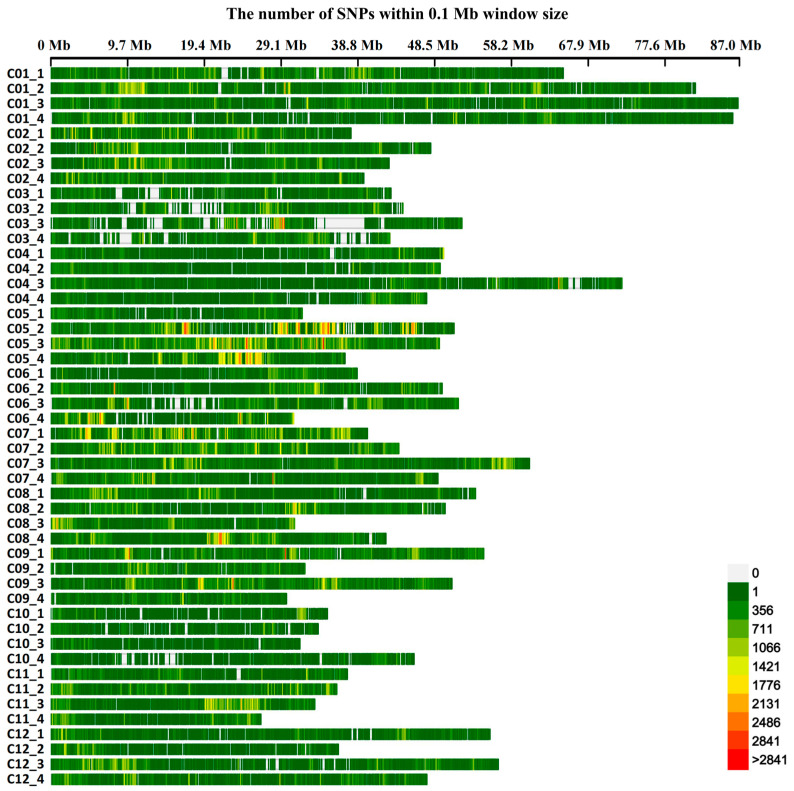
SNP distributions on the 48 chromosomes of potato. The horizontal axis displays the chromosome length; the 0–2841 legend insert indicates the SNP density.

We used a total of 4,535,735 high-quality SNPs for a GWAS of the plant height trait, with an average marker density of 2031 SNP/Mb at the genome-wide scale (Table S2). The lowest marker density (608 SNP/Mb) was found on Chr10A4, whereas the highest marker density (5061 SNP/Mb) was found on Chr05A3. Thus, the markers were unevenly distributed on the tetraploid potato Q9 genome (Figure 2).

### 3.3. Genome-Wide Association Analysis of Plant Height

**Figure 3:**
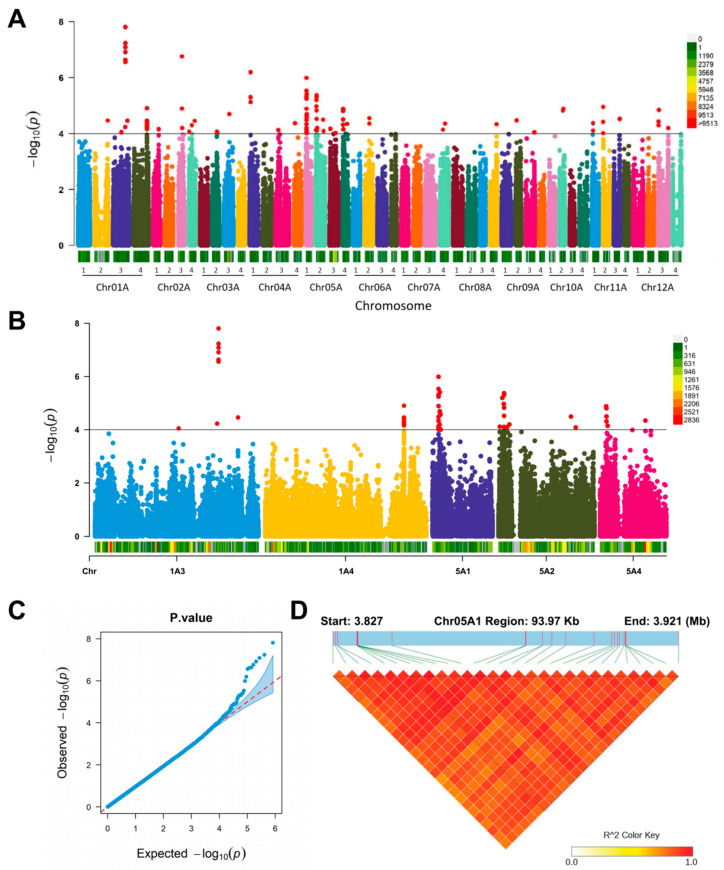
The genome-wide association analysis of plant height. (**A**,**B**) The Manhattan plot of the plant height. Horizontal axis displays the chromosome: 0–9513 legend insert indicates the SNP density (**A**) and 0–2836 legend insert indicates the SNP density (**B**); (**C**) Quantile–quantile plot for the plant height; (**D**) the LD heat map of the plant height associated region on Chr05A1; Red and white color legend insert indicate high and low degree of linkage disequilibrium, respectively.

We used GAPIT to perform GWAS analysis on the phenotypic and genotypic data of the potato plant height using an MLM and plotted the Manhattan and Q-Q plots. Subsequently, we obtained a total of 92 significant SNP loci (Table S3) and found that the significant SNPs had a certain distribution pattern on chromosomes 1, 2, 3, 4, 5, 11, and 12 (Figure 3A), with A3 and A4 on chromosome 1 and A1, A2, and A4 on chromosome 5 being particularly significant (Figure 3B). Since we speculated that there might be important molecular markers associated with plant height on these two chromosomes, Q-Q plots helped check the reliability of our markers. It found that the model fit was good and the significant SNP loci were significantly higher than the fitted curve (Figure 3C). To further determine the linkage disequilibrium within the association region, we plotted the LD heat map of the A1 haplotype association region of chromosome 5 using PopLDdecay and LDBlockShow and found a very strong association of SNPs in the 38.827–38.921 Mb region (Figure 3D).

### 3.4. Identification and Analysis of Candidate Genes

We identified the upstream and downstream genes as candidate genes and obtained a total of 35 candidate genes via screening and identification. Among them, five were transcription factors (bHLH, MYB, TALE, ERF, and B3) (Table S4). To better understand the functions of the candidate genes, we performed their KEGG enrichment analysis and found that “plant hormone signal transduction” had the most candidates, followed by “metabolic pathways” (Figure S1). Additionally, they were also enriched in “zeatin biosynthesis”, “starch and sucrose metabolism”, and the “MAPK (mitogen-activated protein kinase) signaling pathway.” Candidate genes include the *FAR1* gene, *methyltransferase*, *ethylene response factor*, and *ubiquitin protein ligase*, which may be related to potato plant height regulation.

**Figure 4:**
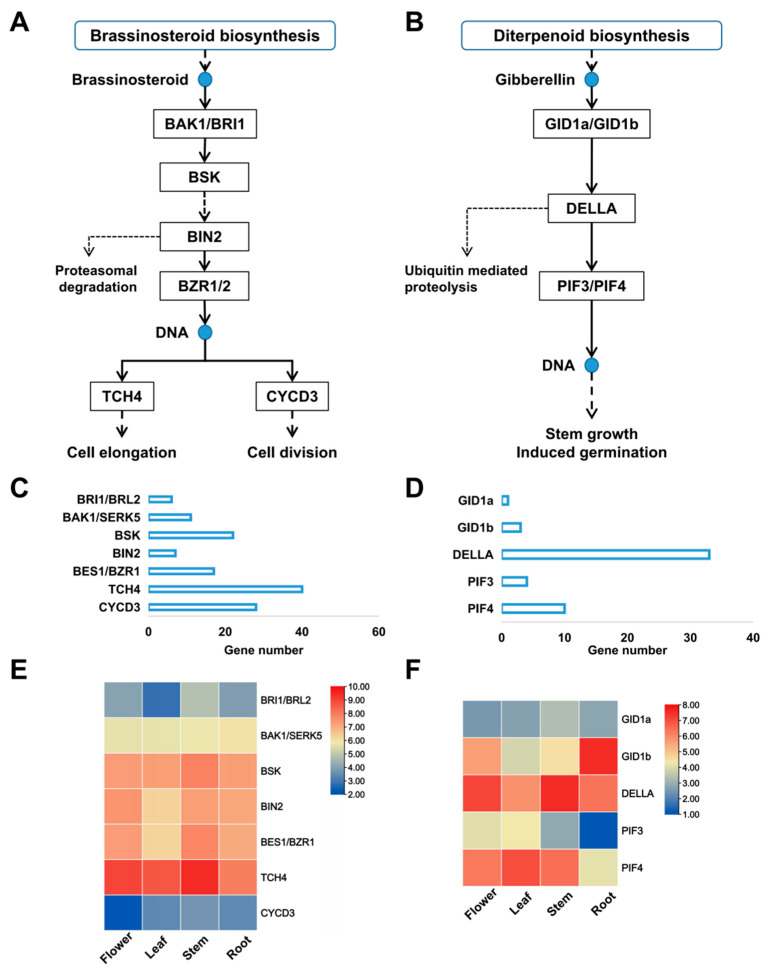
The candidate genes in potato brassinolide and gibberellin pathways. (**A**) potato-brassinolide pathway. (**B**) potato gibberellin pathway. (**C**,**D**) The copy number of candidate genes in potato brassinolide (**C**) and gibberellin (**D**) pathways. (**E**,**F**) Expression profiles of associated, differentially expressed candidate genes among different tissues in potatoes.

Upon further analysis, we identified six genes in the phytohormone signal transduction pathway, mainly *gibberellin receptor 1* (*GID1*) and transcription factor *PIF3* of the GA signaling pathway and *BR-signaling kinase* (*BSK*) and *D-type cyclin family 3* (*CYCD3*) of the BR signaling pathway. To further resolve the genes related to gibberellin and brassinolide in potatoes, we identified the genes related to the tetraploid potato genome and mapped their regulatory pathways (Figure 4A,B). After counting, we found that there were 131 genes in the BRs signaling pathway, of which *xyloglucosyl transferase* (*TCH4*) and *CYCD3* were the most abundant (Figure 4C) and widely distributed in the genome (Table S5). Additionally, they may be related to regulating cell elongation and division processes. The gibberellin signaling pathway has 51 genes, among which *DELLA* is the most abundant, followed by *PIF4*, *PIF3*, and *GID1* (Figure 4D). We found that the *DELLA*, *PIF3* (Phytochrome interacting factor 3), and *GID1* are distributed in chromosome 1, with *PIF3* especially being present only in chromosome 1 and GID1a being present only in the A3 haplotype of chromosome 1 (Table S6, Figure S2).

We also analyzed the gene expression profile in the flowers, stems, leaves, and roots of the tetraploid potato cultivar Q9. The results showed that the expression of *TCH4*, *BSK*, and *CYCD3* in BR signaling was significantly higher in the stems than in the other tissues (Figure 4E). The *GID1a*/*GID1b* expression in the gibberellin signaling pathway was highest in the roots, with *DELLA* being the highest in the stems and flowers. Moreover, *PIF3* and *PIF4* were the most highly expressed in the leaves.

## 4. Discussion

The genetic gain in potatoes has been small as compared to other major crops, with the tetraploid inheritance complexity being a key factor hindering the genetic improvement of cultivated potatoes [32]. With the rapid development of sequencing technology, the genomes of different potato varieties and their ploidy levels were gradually deciphered, especially the assembly of the genomes of tetraploid cultivated potatoes Otava [33], C88 [34], and Q9 [35]. This laid the groundwork for understanding the genetic mechanism of tetraploid potatoes, which is crucial in breeding common tetraploid cultivated potato varieties. In this study, we re-sequenced 370 varieties or strains of tetraploid potatoes, performed the GWAS analysis for the plant height traits using the tetraploid potato genome as a reference, mined for significant molecular markers, and analyzed the candidate genes.

Plant height is an important component of its varietal structure, and its alteration can not only improve the plant’s stress adaptation ability but also improve its yields. Since the Green Revolution in the 1950s, great success has been achieved in reducing plant height to improve actual crop yields [5]. In this study, GWAS analysis of plant height traits revealed the presence of significant markers on multiple chromosomes that may be associated with the regulation of plant height. Among them, especially the A3 haplotype of chromosome 1, we found *PIF3* and *GID1* at the significant locus attachment. In active GA signaling in plants, the gibberellin-insensitive dwarf gene *GID1* (*gibberellin insensitive dwarf 1*) encodes a gibberellin-binding receptor that causes the DELLA protein to aggregate into the SCF^SLY1^ complex, thus resulting in ubiquitin-mediated degradation of DELLA protein, which activates its downstream signaling and affects the plant growth and development [36]. The phytochrome-interacting factors (PIFs) are a class of bHLH transcription factors that were first identified and shown to regulate photomorphogenesis [37]. PIFs are thought to be hubs that integrate both external environmental factors and internal signals during plant development, thereby regulating the expression of their downstream genes [38]. Thus, these are essential genes in the GAs transduction pathway, which finely regulates the entire transcriptome network. Research shows that GA metabolism and signaling are both critical for controlling plant height, which is consistent with the results of this study [9].

Additionally, we identified one *BSK* gene and three *CYCD3* genes on the A3 haplotype of chromosomes 1 and 2. *BSK* and *CYCD3* are important components of the BR signal pathway. Studies have shown that *BSK* plays a crucial role in plant growth, development, and stress regulation [39]. *CYCD3* can promote plant growth by controlling cell division [40,41]. We speculate that *BSK* and *CYCD3* can further control the establishment of plant height by regulating BRs. BRs also interact with other plant signaling pathways, notably those of light and the hormones auxin, GA, abscisic acid (ABA), and ethylene [42]. We found that the *far-red impaired response 1* (*FAR1*) may be involved in the regulation of plant height [43]. The protein encoded contains a DNA-binding domain and belongs to a transposase-derived class of transcription factors that directly activate the expression of the far-red light gene *FHY1*/*FHL*, an important regulatory player in the plant starch anabolism and energy deprivation processes triggered by carbon starvation [43]. Sugars play an important role in plant height development by providing the necessary raw materials for cell division and elongation, like the cloned plant height trait regulator gene *SXD1*. Its mutant leaves do not export sucrose properly, thereby resulting in dwarf plants [21].

Furthermore, some transcription factors may be involved in plant height development, and candidate genes include *MYB* and *ERF*. The MYB transcription factors are a powerful superfamily of transcription factors in plants, and recent studies have shown that they play an important regulatory role in plant growth and development [44,45]. Overexpression of MYB has been found to alter the plant structure, as evident from the increased lateral branching and reduced plant height [46]. The genes *GmGAMYB* [47] and *GmLHY* [48] regulate plant height via the gibberellin pathway, where *GmGAMYB* is induced by gibberellin to up-regulate *GmGA20ox* expression, which increases the plant height. Moreover, *GmLHY* regulates plant height by directly or indirectly increasing the expression levels of GA synthesis-related and GA reaction-related genes.

In this study, a GWAS of plant height traits in 370 cultivars of tetraploid potatoes revealed that the most significant molecular markers associated with potato plant height were located on chromosome 1, while the more significant molecular markers were also present on chromosomes 2, 4, and 5. Additionally, we identified 35 candidate genes, six of which were related to phytohormone signaling, especially GAs and BRs. Therefore, these molecular markers and candidate genes will provide good markers and genetic resources for potato plant height regulation, thus enhancing its breeding process.

## Figures and Tables

**Table 1:** Plant height index standard in potato germplasms.

Agronomic Trait		The Name of Standard Cultivar	Height Index
Plant height	Extremely short	A-6	1
	Very short to short	-	2
	Short	Dongnong303	3
	Short to medium	-	4
	Medium	Kexin2	5
	Medium to high	-	6
	High	Hongtudou	7
	High to very high	-	8
	Very high	Qingshu9 (Q9)	9

**Table 2:** Summary of SNPs and indels among the 370 tetraploid potato cultivars.

	SNPs				Indels			
	A1	A2	A3	A4	A1	A2	A3	A4
Chr01	138,006	194,142	129,323	127,535	15,004	17,111	10,826	14,762
Chr02	102,837	101,023	117,660	85,563	10,583	9856	12,222	9275
Chr03	55,117	76,170	85,021	68,587	6313	7950	6530	9677
Chr04	57,575	57,327	86,486	58,568	7218	6798	8429	6109
Chr05	44,400	197,492	249,222	136,165	6255	14,701	22,302	11,520
Chr06	47,830	91,815	98,615	72,776	6701	8380	10,860	7075
Chr07	186,990	158,514	140,408	105,042	15,751	12,262	11,911	11,757
Chr08	99,099	101,120	73,798	116,424	8699	11,011	7217	10,402
Chr09	142,118	70,221	150,275	47,334	12,861	8067	13,384	5101
Chr10	29,802	26,892	21,862	28,140	3480	3489	2049	2904
Chr11	53,935	81,915	96,253	48,863	6439	10,028	10,263	6931
Chr12	61,094	38,639	97,251	80,491	5612	4649	11,682	8549

## Data Availability

Supporting data can be found at the National Genomics Data Center and are accessible at http://bigd.big.ac.cn/ (accessed on 22 March 2022) under BioProject numbers PRJCA011806.

## Supplementary Materials

The following supporting information can be downloaded at: https://www.mdpi.com/article/10.3390/genes14020507/s1. Table S1: Identification of 370 tetraploid potatoes’ origin and their plant heights; Table S2: Density statistics of SNPs on the tetraploid potato chromosomes; Table S3: Potato plant height association analysis of the significant SNP sites; Table S4: Candidate genes for the significant association markers; Table S5: The number of genes involved in the BR signal transduction pathway and their host chromosomes; Table S6: The number of genes involved in the GAs signal transduction pathway and their host chromosomes; Figure S1: KEGG enrichment analysis of the candidate genes; Figure S2: Genes associated with the gibberellin signaling transduction pathway and their chromosomal distribution.
