# Supplementary material for: Insights into the Genetic Determination of the Autotetraploid Potato Plant Height
Source: Genes (Basel). 2023 Feb 16;14(2):507. doi: 10.3390/genes14020507 (PMC9957462; doi:10.3390/genes14020507)
Supplement: Supplementary file 1 [file genes-14-00507-s001.zip › genes-2151685-supplementary.pdf]

Table S1 Identification of 370 tetraploid potato origin and their plant heights

Number	Name	Sourse	Plant height	Number	Name	Sourse	Plant height
ST_1_10	124	unknown	7	ST_4_12	5p1-1	China	3
ST_1_12	11	unknown	5	ST_4_13	Russ2	Russia	2
ST_1_13	Katahdin×ke	China	3	ST_4_14	DR-4	China	4
ST_1_14	Ke5-11	China	5	ST_4_16	Feixing.2	unknown	5
ST_1_15	e5-1	Peru	5	ST_4_17	Shiyin08—1	unknown	5
ST_1_16	R16	Peru	4	ST_4_18	E8	Peru	5
ST_1_17	Hua525.1	China	4	ST_4_2	Perou08-2	Peru	6
ST_1_18	Jia7	Canada	3	ST_4_20	393882.44	Peru	7
ST_1_21	Zhong9	China	1	ST_4_21	Cy-II-9	Peru	4
ST_1_22	DM	unknown	4	ST_4_22	UP-3	China	3
ST_1_23	Ke9912-3	China	1	ST_4_23	DR-3	China	4
ST_1_26	Ke200373-13	China	3	ST_4_25	manana	unknown	3
ST_1_27	Qing5.1	China	3	ST_4_26	139	unknown	6
ST_1_28	Ke8	China	3	ST_4_27	Norship	unknown	1
ST_1_29	720018.1	unknown	3	ST_4_28	Jiadabai	Canada	1
ST_1_3	Zhong227	China	2	ST_4_29	E19.1	Peru	5
ST_1_31	B18.1	Peru	4	ST_4_3	E65	Peru	6
ST_1_34	B11	Peru	4	ST_4_30	Fuke76	China	4
ST_1_35	Isread2.1	Israel	5	ST_4_31	IP84008.4.1	Peru	5
ST_1_36	CIP09-6	Peru	5	ST_4_33	Youxiao	unknown	2
ST_1_37	BH2-5	China	5	ST_4_36	Israel	Israel	5
ST_1_38	E12.1	Peru	5	ST_4_37	IP84008.4.2	Peru	7
ST_1_39	F20	Peru	4	ST_4_38	Long8	China	2
ST_1_4	CIP09-2	Peru	3	ST_4_39	ACD338	unknown	2
ST_1_40	Isread1	Israel	5	ST_4_4	183	unknown	3
ST_1_41	5p2-1	China	4	ST_4_40	Isread2.2	Israel	1
ST_1_42	B5	Peru	3	ST_4_41	UK3	unknown	3
ST_1_44	DR-9.1	China	4	ST_4_43	Cy-III-18	Peru	1
ST_1_45	BE13-7	Russia	3	ST_4_44	DY4-5	China	2
ST_1_46	B1	Peru	6	ST_4_45	E80	Peru	4
ST_1_47	B3	Peru	5	ST_4_46	Kajin	China	3
ST_1_48	RH	unknown	4	ST_4_47	E76	Peru	5
ST_1_5	Ke6108-17	China	2	ST_4_48	2340	unknown	4
ST_1_50	BE13-4	Russia	5	ST_4_49	IP84008.3	Peru	6
ST_1_53	DR-6.1	China	4	ST_4_5	348	unknown	3
ST_1_55	5P2-6.1	China	4	ST_4_51	Jemsay	China	2
ST_1_57	T1	Peru	5	ST_4_52	NZY	New Zealand	3
ST_1_58	Zhongshu227	China	6	ST_4_53	758792	Peru	3
ST_1_59	283	unknown	3	ST_4_54	F8701.2	China	2
ST_1_61	ZhongC901	China	4	ST_4_56	268	unknown	4

ST_1_62	B14.1	Peru	4	ST_4_57	E14.1	Peru	3
ST_1_63	Qing10	China	4	ST_4_58	C88	China	2
ST_1_64	Huayun	unknown	6	ST_4_6	E22	Peru	7
ST_1_65	CIP09-16	Peru	5	ST_4_61	Hermes	unknown	2
ST_1_67	5p2-3	China	3	ST_4_62	LC-98	unknown	2
ST_1_68	T2	Peru	4	ST_4_63	Eromnede	unknown	2
ST_1_69	CIP09-10	Peru	6	ST_4_64	BDRA	unknown	4
ST_1_7	Ningshu1.1	China	3	ST_4_65	Zhukefu×FL1867	China	2
ST_1_70	NE303.1	China	3	ST_4_67	63	unknown	3
ST_1_71	R1R3.1	Peru	5	ST_4_7	AKCFC	unknown	6
ST_1_73	W	Peru	4	ST_4_72	Fedori	unknown	3
ST_1_75	B19	Peru	2	ST_4_73	4(2012)	unknown	2
ST_1_76	E62	Peru	6	ST_4_8	R9.1	Peru	3
ST_1_77	D8	Peru	3	ST_4_83	Pepo416.2	Peru	3
ST_1_79	CIP09-27	Peru	3	ST_4_85	R10	Peru	5
ST_1_8	Jklway	Peru	1	ST_4_86	Xingjia1	China	2
ST_1_9	CIP09-18	Peru	5	ST_4_88	D13	Peru	4
ST_2_1	R3R4	Peru	5	ST_4_89	JD202461-199	China	2
ST_2_10	Yunshu501	China	6	ST_4_9	288	unknown	6
ST_2_14	Rigao	unknown	3	ST_4_91	P5.1	Australia	4
ST_2_15	D23	Peru	5	ST_4_92	R1R2	Peru	5
ST_2_16	Jizhangshu8	China	6	ST_4_93	Supiror	China	3
ST_2_17	5p2-6.2	China	6	ST_4_94	Jia3	Canada	3
ST_2_18	4p2-2	China	5	ST_4_95	5P1-5	China	4
ST_2_19	Ke6	China	5	ST_4_97	UK6	unknown	3
ST_2_2	D16	Peru	4	ST_4_98	Hongwenbai	United States	3
ST_2_20	Ke3	China	4	ST_4_99	UK12	unknown	3
ST_2_21	Zhongshu19	China	3	ST_5_1	DT033	China	5
ST_2_22	10	China	4	ST_5_10	E12.3	Peru	6
ST_2_24	R2	Peru	4	ST_5_11	W15	Peru	6
ST_2_25	Zhongshu18	China	5	ST_5_12	BoII.2	unknown	2
ST_2_26	Bulaodao	China	5	ST_5_13	DT02	China	5
ST_2_27	Maiken1	China	4	ST_5_14	A2.1	Peru	5
ST_2_28	Shenyanwo	China	6	ST_5_15	Ninglu0.1	China	4
ST_2_3	Ke21	China	3	ST_5_16	Qing05-12-6	China	6
ST_2_30	W5	Peru	5	ST_5_17	Ningshu1.2	China	5
ST_2_31	Feixing.1	unknown	4	ST_5_18	BoS	China	6
ST_2_32	Cy-II-19	Peru	3	ST_5_19	DR-6.2	China	5
ST_2_33	B10	Peru	5	ST_5_2	DR-9.2	China	5
ST_2_34	E52	Peru	6	ST_5_20	LT-7	China	2
ST_2_35	B16	Peru	6	ST_5_21	BH2-4	China	3
ST_2_39	F21	Peru	5	ST_5_22	Hu9052—2	China	5

ST_2_4	BoII.1	unknown	3	ST_5_23	Fuke212	China	3
ST_2_40	B4.1	Peru	4	ST_5_24	E60	Peru	7
ST_2_41	Zhengshu8	China	3	ST_5_26	Cy-III-19	Peru	2
ST_2_43	11(Belleile)	unknown	5	ST_5_28	R13	Peru	2
ST_2_44	F12	Peru	2	ST_5_29	ACD342	unknown	3
ST_2_45	B6	Peru	5	ST_5_3	CIP09-5	Peru	4
ST_2_46	B24	Peru	4	ST_5_30	T6.2	unknown	4
ST_2_48	8	China	5	ST_5_31	258	unknown	3
ST_2_49	E12.2	Peru	7	ST_5_32	B18.2	Peru	3
ST_2_5	Ke200373	China	3	ST_5_33	E20	Peru	3
ST_2_50	B13	Peru	4	ST_5_34	Ning16	China	5
ST_2_52	F17	Peru	4	ST_5_35	E5	Peru	6
ST_2_53	T6.1	Peru	4	ST_5_36	Tuo175	China	5
ST_2_54	NE303.2	China	3	ST_5_37	Zhongshu21	China	3
ST_2_55	B9	Peru	5	ST_5_40	Sebage	China	3
ST_2_56	E99	Peru	7	ST_5_42	S8	Peru	8
ST_2_58	Datongliwaihuang	China	3	ST_5_43	E14.2	Peru	8
ST_2_62	Cy-II-25	Peru	5	ST_5_44	A1	Peru	6
ST_2_63	Jizhangshu3	China	6	ST_5_45	E23	Peru	6
ST_2_65	JK5	Peru	3	ST_5_47	Chinese Red	China	5
ST_2_68	6	China	4	ST_5_5	Hu5（115）	China	3
ST_2_7	P6	Australia	5	ST_5_52	KW-29	Peru	6
ST_2_71	F13	Peru	3	ST_5_53	S17	Peru	5
ST_2_72	CIP09-3	Peru	3	ST_5_56	CIP10-1.2	Peru	6
ST_2_73	5p2-4	China	3	ST_5_57	Hu5	China	3
ST_2_75	CIP09-9	Peru	4	ST_5_58	B2	Peru	6
ST_2_76	F18	Peru	3	ST_5_59	Qingzangxuan2	China	6
ST_2_78	CIP10-1.1	Peru	4	ST_5_6	Minshu2	China	5
ST_2_79	BE13-3	Russia	6	ST_5_60	Yanshu3	China	5
ST_2_8	Bf1-1	Peru	3	ST_5_62	Tian0527-4	China	4
ST_2_80	Qingshu9	China	9	ST_5_63	A2.2	Peru	6
ST_2_9	71	unknown	2	ST_5_65	Dd-I-16	Peru	6
ST_3_1	Cy-III-2	Peru	2	ST_5_67	S1	Peru	5
ST_3_10	2（2012）	unknown	2	ST_5_68	S25.2	Peru	3
ST_3_11	W38	Peru	6	ST_5_69	No Name	unknown	5
ST_3_12	182	unknown	5	ST_5_7	W3	Peru	5
ST_3_13	Pepo416.1	Peru	4	ST_5_70	D11	Peru	4
ST_3_14	4p1-2	China	4	ST_5_72	KW-11	Peru	5
ST_3_15	Zhong4.1	China	3	ST_5_74	S5	Peru	4
ST_3_16	Perou08-3	Peru	6	ST_5_78	S.st	Peru	3
ST_3_17	Dongnong303	China	4	ST_5_79	Ninglu0.2	China	4
ST_3_18	Perou08-1	Peru	6	ST_5_8	Anuile	unknown	4
ST_3_19	F8701.1	China	3	ST_5_81	KW-40	Peru	3

ST_3_2	Zihuabai	China	1	ST_5_82	BE13-11	Russia	1
ST_3_20	Zhong113	China	6	ST_5_84	Y3	Peru	2
ST_3_21	8089-115	unknown	4	ST_5_87	B27	Peru	2
ST_3_22	S25.1	Peru	8	ST_5_88	F4	Peru	3
ST_3_23	Bf1-4	Peru	2	ST_5_89	BH1-1	China	3
ST_3_24	H24	unknown	7	ST_5_9	Nunone	unknown	2
ST_3_25	5p1-4	China	4	ST_5_91	Isread2.3	Israel	2
ST_3_27	Zhong4.2	China	2	ST_5_92	5p2-5	China	2
ST_3_28	720018.2	unknown	2	ST_6_1	Zaodabai.2	China	4
ST_3_29	Shenzhong222	China	2	ST_6_11	Jiabai	Canada	2
ST_3_3	E5-2	Peru	7	ST_6_12	A6	Peru	6
ST_3_31	Bf1-2	Peru	2	ST_6_13	E41	Peru	6
ST_3_33	Qing8	China	6	ST_6_14	R1R3.2	Peru	4
ST_3_35	Dd-II-6	Peru	5	ST_6_15	B14.2	Peru	4
ST_3_36	NTB.1	unknown	7	ST_6_17	E18	Peru	8
ST_3_37	H2	unknown	3	ST_6_18	Eroracade	unknown	3
ST_3_38	Y1	Peru	2	ST_6_19	4p1-3	China	5
ST_3_39	ZhongA9215-84	China	2	ST_6_2	Zhongda1	China	4
ST_3_4	E73.1	Peru	7	ST_6_20	B4.2	Peru	7
ST_3_40	Hu8212-3.1	China	2	ST_6_21	E73.2	Peru	6
ST_3_41	Atlantic	United States	2	ST_6_22	E39	Peru	6
ST_3_43	nonona	unknown	2	ST_6_23	BE13-10	Russia	4
ST_3_47	36221	unknown	3	ST_6_24	E42	Peru	3
ST_3_5	E93	Peru	7	ST_6_25	Taihong	China	2
ST_3_51	IVP101	China	5	ST_6_26	LT-5	China	2
ST_3_52	38909	unknown	3	ST_6_27	Felix Rita	Netherlands	1
ST_3_53	UK9	unknown	5	ST_6_28	A11	Peru	6
ST_3_59	Zaodabai.1	China	4	ST_6_3	Ke1	China	3
ST_3_6	Zangxuan	China	6	ST_6_32	A3	Peru	6
ST_3_60	Hu8212-3.2	China	3	ST_6_33	R9.2	Peru	3
ST_3_61	ACD151	unknown	5	ST_6_34	Zhangshu1	China	6
ST_3_63	Hua525.2	China	3	ST_6_36	E19.2	Peru	6
ST_3_64	Cy-II－7	Peru	6	ST_6_38	Taihe	China	2
ST_3_65	Ｗ２０	Peru	5	ST_6_39	B7	Peru	6
ST_3_66	Bf1-3	Peru	5	ST_6_4	Yan97—7	China	2
ST_3_68	Ningshu0.1	China	4	ST_6_40	KW-59	Peru	7
ST_3_7	NTB.2	unknown	7	ST_6_43	4p2-8.1	China	6
ST_3_70	426	unknown	2	ST_6_45	06-26-7	unknown	6
ST_3_74	210(2012)	unknown	2	ST_6_48	KW-22	Peru	7
ST_3_76	Qing168	China	3	ST_6_49	KW-56	Peru	6
ST_3_77	DB（Df）	unknown	4	ST_6_50	4p2-8.2	China	6
ST_3_8	Chunshu1	China	2	ST_6_51	S115	Peru	7
ST_3_83	Ziyun	unknown	7	ST_6_52	KW-41	Peru	4

ST_3_87	Ke13	China	4	ST_6_53	KW-24	Peru	6
ST_3_88	D568	Peru	5	ST_6_54	UK8	unknown	6
ST_3_89	Qing5.2	China	5	ST_6_57	Dd-II-17	Peru	6
ST_3_9	Jiadahong	Canada	3	ST_6_58	Jin2004-4-14	China	5
ST_3_90	W28	Peru	5	ST_6_63	Dd-II-11	Peru	5
ST_3_91	CIP09-1	Peru	5	ST_6_67	A4	Peru	5
ST_3_92	CIP09-12	Peru	5	ST_6_7	Jiabai7	Canada	4
ST_3_93	UK7	unknown	3	ST_6_8	222	unknown	7
ST_3_94	Maiyi233	unknown	5	ST_6_81	L9901-132	unknown	4
ST_3_95	E66	Peru	4	ST_6_83	D4	Peru	3
ST_3_96	Qingshu3	China	2	ST_6_84	P5.2	Australia	4
ST_3_97	Weishu1	China	2	ST_6_85	BH2-1.1	China	6
ST_3_98	4P2-9	China	4	ST_6_86	D14	Peru	7
ST_3_99	D12	Peru	5	ST_6_88	BH2-1.2	China	2
ST_4_1	Dinila	unknown	5	ST_6_89	Isread3	Israel	4
ST_4_10	DR-8	China	4	ST_6_9	Bai5-2	China	3
ST_4_11	E94	Peru	7	ST_CH_3	CH_3	Peru	4

Table S2 Density statistics of SNPs on the tetraploid potato chromosomes

	SNPs	length (bp)	marker density (Mb/SNP)
Chr01A1	138,006	64,903,345	2,126
Chr01A2	194,142	81,529,071	2,381
Chr01A3	129,323	86,866,244	1,489
Chr01A4	127,535	86,198,547	1,480
Chr02A1	102,837	37,955,832	2,709
Chr02A2	101,023	48,074,756	2,101
Chr02A3	117,660	42,785,532	2,750
Chr02A4	85,563	39,579,123	2,162
Chr03A1	55,117	43,048,385	1,280
Chr03A2	76,170	44,510,147	1,711
Chr03A3	85,021	51,999,378	1,635
Chr03A4	68,587	42,884,546	1,599
Chr04A1	57,575	49,683,045	1,159
Chr04A2	57,327	49,251,488	1,164
Chr04A3	86,486	72,185,234	1,198
Chr04A4	58,568	47,521,664	1,232
Chr05A1	44,400	31,841,068	1,394
Chr05A2	197,492	51,077,589	3,867
Chr05A3	249,222	49,243,048	5,061
Chr05A4	136,165	37,238,887	3,657
Chr06A1	47,830	38,746,277	1,234
Chr06A2	91,815	49,515,538	1,854
Chr06A3	98,615	51,513,620	1,914
Chr06A4	72,776	30,700,426	2,371

Chr07A1	186,990	40,049,978	4,669
Chr07A2	158,514	43,998,597	3,603
Chr07A3	140,408	60,568,584	2,318
Chr07A4	105,042	48,941,444	2,146
Chr08A1	99,099	53,685,969	1,846
Chr08A2	101,120	50,070,439	2,020
Chr08A3	73,798	31,008,315	2,380
Chr08A4	116,424	42,454,690	2,742
Chr09A1	142,118	54,755,689	2,595
Chr09A2	70,221	32,249,479	2,177
Chr09A3	150,275	50,775,432	2,960
Chr09A4	47,334	29,801,604	1,588
Chr10A1	29,802	35,118,964	849
Chr10A2	26,892	33,871,578	794
Chr10A3	21,862	31,575,115	692
Chr10A4	28,140	46,272,311	608
Chr11A1	53,935	37,496,597	1,438
Chr11A2	81,915	36,170,018	2,265
Chr11A3	96,253	33,547,497	2,869
Chr11A4	48,863	26,668,463	1,832
Chr12A1	61,094	55,568,083	1,099
Chr12A2	38,639	36,358,531	1,063
Chr12A3	97,251	56,547,829	1,720
Chr12A4	80,491	47,543,799	1,693

Table S3 Potato plant height association analysis of the significant SNP sites

Chromosome	Position	P value	Chromosome	Position	P value
Chr01A2	73,244,126	0.0000341	Chr05A1	3,938,663	0.0000202
Chr01A3	44,276,715	0.0000889	Chr05A1	3,938,717	0.0000226
Chr01A3	64,623,868	0.000059	Chr05A1	3,938,776	0.0000925
Chr01A3	65,361,191	0.000000275	Chr05A1	3,939,031	0.00000388
Chr01A3	65,361,213	0.000000243	Chr05A1	4,305,460	0.000027
Chr01A3	65,361,282	8.17E-08	Chr05A1	4,411,647	0.0000993
Chr01A3	65,364,494	0.000000122	Chr05A2	2,258,006	0.00000636
Chr01A3	65,364,558	1.56E-08	Chr05A2	3,023,030	0.0000107
Chr01A3	65,364,585	5.82E-08	Chr05A2	3,178,958	0.0000786
Chr01A3	65,364,640	0.000000234	Chr05A2	3,206,459	0.0000302
Chr01A3	75,661,144	0.0000346	Chr05A2	3,209,158	0.0000149
Chr01A4	73,885,250	0.0000385	Chr05A2	3,216,208	0.00000422
Chr01A4	73,885,292	0.0000346	Chr05A2	3,216,363	0.00000489
Chr01A4	73,886,127	0.0000583	Chr05A2	38,544,157	0.000032
Chr01A4	73,901,099	0.0000457	Chr05A2	41,067,589	0.0000821
Chr01A4	73,901,106	0.0000536	Chr05A2	4,843,193	0.0000795
Chr01A4	73,901,161	0.0000647	Chr05A2	5,990,666	0.0000633

Chr01A4	73,901,168	0.0000125	Chr05A2	957,753	0.0000767
Chr01A4	73,902,576	0.000069	Chr05A3	33,012,332	0.0000955
Chr02A1	28,660,758	0.0000696	Chr05A3	5,638,765	0.0000676
Chr02A3	23,891,060	0.000000175	Chr05A4	24,010,545	0.0000451
Chr02A3	23,891,217	0.0000128	Chr05A4	3,289,734	0.0000132
Chr02A3	24,974,425	0.0000639	Chr05A4	3,430,079	0.0000156
Chr02A4	16,645,713	0.0000507	Chr05A4	3,441,654	0.0000316
Chr02A4	2,478,700	0.0000858	Chr05A4	3,441,928	0.0000484
Chr02A4	31,737,074	0.0000355	Chr05A4	3,441,934	0.0000484
Chr03A2	25,479,946	0.0000873	Chr05A4	4,050,955	0.0000713
Chr03A2	27,536,749	0.0000889	Chr06A2	26,458,246	0.0000284
Chr03A3	29,477,090	0.0000201	Chr06A2	26,458,266	0.0000439
Chr03A3	29,477,091	0.0000201	Chr07A4	16,662,222	0.000073
Chr04A1	5,318,931	0.00000502	Chr07A4	28,364,058	0.0000436
Chr04A1	5,318,941	0.000000641	Chr08A4	37,288,734	0.0000462
Chr04A1	5,318,955	0.00000746	Chr09A2	4,899,622	0.000034
Chr04A3	14,148,959	0.0000756	Chr09A3	46,194,745	0.0000908
Chr04A4	10,345,294	0.0000428	Chr10A2	19,298,458	0.000015
Chr05A1	3,257,038	0.0000465	Chr10A2	21,414,780	0.0000129
Chr05A1	3,257,113	0.0000744	Chr11A1	4,821,383	0.0000788
Chr05A1	3,257,119	0.0000892	Chr11A1	5,141,276	0.0000428
Chr05A1	3,257,130	0.00000102	Chr11A2	1,022,973	0.0000975
Chr05A1	3,257,147	0.00000567	Chr11A2	1,023,032	0.0000112
Chr05A1	3,257,160	0.0000329	Chr11A2	1,023,059	0.0000388
Chr05A1	3,257,168	0.000097	Chr11A3	31,181,030	0.0000299
Chr05A1	3,257,239	0.00000487	Chr12A3	2,412,232	0.0000507
Chr05A1	3,257,260	0.0000129	Chr12A3	2,412,301	0.0000374
Chr05A1	3,257,402	0.00000294	Chr12A3	3,786,963	0.0000144
Chr05A1	3,822,489	0.0000639	Chr12A3	54,261,742	0.0000641

Table S4 Candidate genes of the significant association markers

Candidate gene	Chr.	Start	End	Description
Soltu.Q9.Chr01A30002275.g	Chr01A3	68,815,258	68,822,588	BR-signaling kinase
Soltu.Q9.Chr01A30002906.g	Chr01A3	75,648,043	75,658,213	NUFIP1
Soltu.Q9.Chr01A30002907.g	Chr01A3	75,667,683	75,673,898	ATPase (P-type)
Soltu.Q9.Chr01A30003072.g	Chr01A3	77,198,530	77,201,638	gibberellin receptor 1 (GID1)
Soltu.Q9.Chr01A30003220.g	Chr01A3	78,799,369	78,804,942	PIF3 (bHLH)
Soltu.Q9.Chr02A30005102.g	Chr02A3	23,355,105	23,356,806	CYCD3
Soltu.Q9.Chr02A30005105.g	Chr02A3	23,394,832	23,395,110	CYCD3
Soltu.Q9.Chr02A30005106.g	Chr02A3	23,409,866	23,411,506	CYCD3
Soltu.Q9.Chr02A30005133.g	Chr02A3	23,890,161	23,892,408	Lipolytic acyl hydrolase (LAH)
Soltu.Q9.Chr02A30005134.g	Chr02A3	23,892,733	23,897,941	Mitogen-activated protein kinase
Soltu.Q9.Chr03A20008670.g	Chr03A2	25,477,511	25,478,453	Protein FAR1-RELATED SEQUENCE
Soltu.Q9.Chr03A20008671.g	Chr03A2	25,478,607	25,478,801	Protein FAR1-RELATED SEQUENCE

Soltu.Q9.Chr04A10010124.g	Chr04A1	5,313,706	5,319,513	maltase-glucoamylase
Soltu.Q9.Chr04A10010125.g	Chr04A1	5,326,819	5,333,560	GHMP kinases N terminal domain
Soltu.Q9.Chr05A10012744.g	Chr05A1	3,264,876	3,265,352	F-box LRR-repeat protein
Soltu.Q9.Chr05A10012841.g	Chr05A1	3,923,867	3,930,253	Resistance protein
Soltu.Q9.Chr05A10012842.g	Chr05A1	3,945,224	3,948,512	May act as a component of the auxin efflux carrier
Soltu.Q9.Chr05A10012869.g	Chr05A1	4,294,669	4,297,148	Rare lipoprotein A (RlpA)-like double-psi beta-barrel
Soltu.Q9.Chr05A10012880.g	Chr05A1	4,410,439	4,413,129	Myb-like DNA-binding domain (MYB)
Soltu.Q9.Chr05A20012774.g	Chr05A2	949,976	950,977	Sulfotransferase 1 family
Soltu.Q9.Chr05A20012775.g	Chr05A2	964,984	965,489	Sulfotransferase 1 family
Soltu.Q9.Chr05A20012959.g	Chr05A2	3,187,376	3,189,844	Heat shock 70 kDa protein
Soltu.Q9.Chr05A20012962.g	Chr05A2	3,213,598	3,228,898	IPP transferase
Soltu.Q9.Chr05A20013150.g	Chr05A2	4,828,987	4,840,093	GDP-fucose protein O-fucosyltransferase
Soltu.Q9.Chr05A20014473.g	Chr05A2	41,063,583	41,064,994	SH3 domain-containing protein
Soltu.Q9.Chr05A40013600.g	Chr05A4	3,290,618	3,293,083	Rare lipoprotein A (RlpA)-like double-psi beta-barrel
Soltu.Q9.Chr05A40013613.g	Chr05A4	3,420,676	3,422,004	methyltransferase activity
Soltu.Q9.Chr05A40013614.g	Chr05A4	3,426,494	3,431,698	GDA1/CD39 (nucleoside phosphatase) family
Soltu.Q9.Chr11A10026323.g	Chr11A1	4,818,246	4,820,953	Zinc-binding dehydrogenase
Soltu.Q9.Chr11A10026357.g	Chr11A1	5,109,532	5,148,813	Toll - interleukin 1 - resistance
Soltu.Q9.Chr11A30029381.g	Chr11A3	31,172,119	31,176,357	BEL1-like homeodomain 8 (TALE)
Soltu.Q9.Chr12A30029797.g	Chr12A3	2,414,018	2,415,576	ethylene-responsive transcription factor (ERF)
Soltu.Q9.Chr12A30029935.g	Chr12A3	3,777,344	3,782,098	Lactoylglutathione lyase
Soltu.Q9.Chr12A30029936.g	Chr12A3	3,782,879	3,783,571	B3 domain-containing protein (B3)
Soltu.Q9.Chr12A30029937.g	Chr12A3	3,787,812	3,792,646	Ubiquitin-protein ligase

Table S5 The number of genes involved in the BR signal transduction pathway and their host chromosomes.

Name	Number	Chromosome
TCH4	40	3A1, 3A2, 3A4, 5A1, 5A3, 5A4, 7A1, 7A2, 7A3, 7A4, 12A2, 12A3, 12A4
CYCD3	28	1A1, 1A2, 1A3, 1A4, 2A1, 2A2, 2A3, 2A4, 4A1, 4A3, 12A3, 12A4
BSK	22	1A1, 1A3, 1A4, 4A2, 4A4, 6A1, 6A2, 6A3, 9A2, 9A3, 9A4, 10A1, 10A2, 10A3, 10A4, 11A2, 11A3, 12A1, 12A2, 12A3,
BES1/BZR1	17	2A1, 2A2, 2A3, 2A4, 3A2, 3A3, 3A4, 4A2, 4A4, 12A1, 12A3
BIN2	7	2A2, 2A3, 2A4, 3A2, 3A3, 3A4, 7A4
BAK1/SERK5	11	1A1, 1A2, 1A4, 10A3, 10A4,
BRI1/BRL2	6	4A1, 4A2, 4A3

Table S6 The number of genes involved in the GAs signal transduction pathway and their host chromosomes

Name	Number	Chromosome
DELLA protein	33	1A1, 1A2, 1A3, 1A4, 5A3, 6A1, 6A2, 6A3, 10A1, 10A3, 10A4, 11A1, 11A2, 11A3, 11A4, 12A3
PIF4	10	6A2, 6A3, 6A4, 7A1, 7A2, 7A3, 7A4, 9A1
PIF3	4	1A1, 1A2, 1A3, 1A4

GID1a	1	1A3
GID1b	3	6A1, 6A2, 6A4

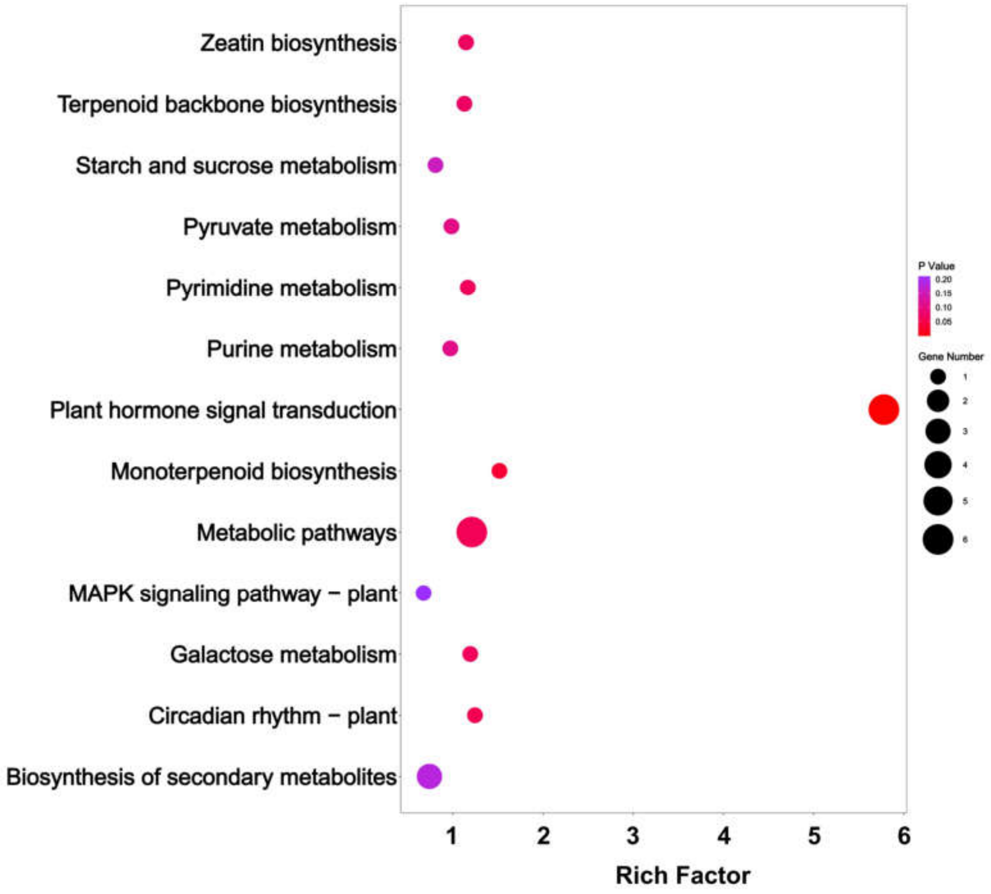

Figure S1 KEGG enrichment analysis of the candidate genes.

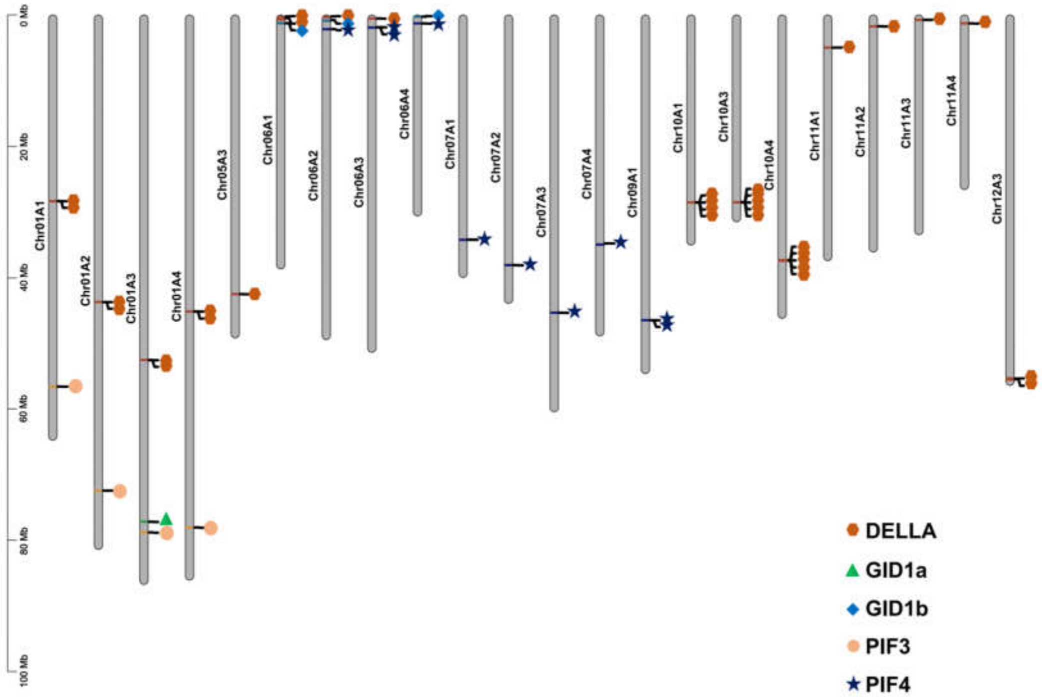

Figure S2: Genes associated with gibberellin signaling transduction pathway and their chromosomal distribution.
